# Transactions Baltimore Pathological Society

**Published:** 1856-12

**Authors:** W. Chew Van Bibber


### EXTRACTS FROM THE TRANSACTIONS OF THE BALTIMORE PATHOLOGICAL SOCIETY.

BY W. CHEW VAN BIBBER, M. D.

### *Croup.*

The subject before the Society being Croup, Dr. Williams spoke as follows:

In the few remarks which I shall now make, I shall say nothing about the symptoms of croup, but shall confine myself strictly to the question of its nature and treatment.

What is the nature of croup? So far as our present knowledge extends, I feel justified in defining it to be a specific inflammation of the air passages, and more especially of the larynx, which is attended by an exudation of false membrane. This exudation may take place in the pharynx, and then extend to the larynx; or it may commence in the larynx, and thence be propagated to surrounding parts. In the former case, it has received the name of *diptheretic croup*; in the latter it is called *pseudo-membranous croup*. For practical purposes, however, so far as treatment is concerned, they may be regarded as the same disease.

What, then, is its treatment?

In view of the specific character of the disease, specific treatment has been proposed. But unfortunately, such treatment has not proved successful.

It only remains to enquire into the indications to be fulfilled by "rational" treatment! What are they?

1st. To prevent the formation of false membranes.

2nd. To cause their expulsion when formed.

3rd. To prevent their reformation.

These objects may be accomplished either by *local* or by *general treatment;* or by both combined.

Local treatment consists in the application of caustics directly to the affected parts. The best caustic seems to be *nitrate of silver*, (20 or 30 grains to an ounce of water,) which must be repeated as often as the violence of the case requires.

The general treatment should consist in the use of bleeding, calomel and emetics, either singly or combined. Old authors are nearly unanimous in recommending blood letting. Indeed, they make it the essential part of their treatment. Recent experimenters, however, are opposed to it. Meigs thinks that it may be used in those cases where the disease begins in the larynx. Calomel has more advocates; and it certainly is a most beneficial remedy; but it is too slow in its operation, to justify us in relying upon it alone. It should always be associated with something more prompt in its action. The best combination, so far as my observation and experience extend, is calomel with emetics. This combination fulfills all the indications of treatment. The calomel, by its defibrinizing qualities, tends to prevent the formation of false membranes—or, if they be already formed, it tends to prevent their spreading to surrounding parts with the same rapidity that they might otherwise assume. The emetics tend to produce their detachment and expulsion.

But whatever may be the theory—practice sanctions the treatment. Meigs mentions eight cases thus treated, of which five recovered. Valleix refers to fifty-three cases, of which thirty-one were treated with emetics and (generally with) calomel. Of these, fifteen recovered.

Twenty-two (of the 53) were treated without emetics, or at least they formed but a secondary part of the treatment. Of these all died but one. All of those that recovered expelled more or less false membrane.

Being thus satisfied of the utility of emetics, the question arises, what emetic should be used? Ipecac. is liable to the objection of losing much of its effect, if its use be continued any length of time, while antimony and sulphate of copper are so irritant as to expose to the risk of producing derangement if not inflammation of the stomach. Alum is free from both these objections, and hence is a most serviceable remedy.

Thus, as a summary of what has been said, we find that the best treatment for croup is a combination of calomel and alum—the latter to be given two or three times a day, so as to produce

*active emesis;* and then during the intervals to administer calomel every one or two hours, so as to bring the patient under its influence as speedily as possible. To this treatment may be added, in many cases, cauterization, and sometimes, though rarely, the use of the lancet.

Should all these means fail, there remains but one remedy more, viz: tracheotomy.

Properly to decide upon the propriety of this operation, we must examine two points, which can only be determined by an appeal to facts, that is, to the result of experiment. These points are the results derived from the operation, and the danger attending it.

1st. Is tracheotomy attended with danger to the life of the patient?

Where it has been performed for the removal of foreign bodies, it has been almost always successful. Prof. H. H. Smith, of Philadelphia, has collected twenty-nine cases, and of this number only one resulted fatally! Prof. Pancoast corroborates this satement, and asserts that he has never seen a fatal case, where the death was clearly assignable to the operation. And such is the experience of French practitioners, so far as I have seen and read.

From these facts, it appears that the operation is not attended by danger, when performed under favorable circumstances.

What is the result when performed for croup?

I have only been able to obtain, from reliable sources, reports of 208 cases. Of these, fifty-nine recovered. This is certainly a most favorable result; and taken in connection with the little danger attending the operation for the removal of foreign bodies, seems to constitute a very strong argument in favor of the operation. The strength of the argument is still increased, if we bear in mind the fact that the operation for croup is deferred until a late period, and the death of the patient imminent. It seems to me that such facts afford a strong inducement to perform the operation.

Before resorting to it, however, we should make faithful use of the medical means at our disposal. If they fail, we would certainly feel justified in favor of the operation.

Dr. Wilson presented the heart and lungs of a patient who had died at the Alms-house on the 21st inst. The point of interest was, that the case had been diagnosed phthisis, while the patient died of pneumonia and pericarditis.

The patient, a female, age unknown, was nearly moribund when she entered the hospital. Could give little account of herself. Had been sick for a long time, and had been under medical treatment some ten days. Was greatly emaciated, and all her symptoms those of great prostration. Was so feeble as to be able to stand but a slight physical examination. This gave perfect flatness on percussion at the apex of the right lung, and extending to the sixth rib. Below this the lung became resonant, and so for the left lung. Palpation gave increased vocal tremor at the apex of the right lung. Auscultation gave apparently well marked cavernous respiration, which an autopsy proved to be the result of a dilated bronchus, surrounded by hepatized lung. Bronchophony, amounting to pectoriloquy, resulted from the same conditions; and on coughing, the air passing through liquid in a dilated bronchus, gave the signs of cavernous rales. All these signs were found at the apex of the right lung—nowhere else—and in the absence of aid from previous history, they were considered symptomatic, and sufficiently diagnostic of phthisis. The patient died in 36 hours after admission. Was unable to submit to a second examination of the chest. Attention was not called to the condition of the heart during life.

On examining the preparation, the two layers of the pericardium were adherent for at least two-thirds of their extent, and when separated, the intervening layer of lymph was found to be several lines in thickness, and gave the peculiar "beef tongue" appearance, or that produced by the quick separation of two surfaces covered with butter.

The lungs were crepitant and healthy everywhere, excepting the upper half of the right, which was solidified by red hepatization, the second stage of pneumonia.

Dr. Wilson said, that from his own experience, pneumonia confined to the apex of the lung, was rather of rare occurrence. He could call to mind only two cases at this time, and one of these was associated with tuburcles.

Drs. Murdoch, Pottinger and Van Bibber had seen at the Alms-house many cases in which the pneumonia was confined to the apices of the lungs. During some winters it seemed more common than in others, but from what cause or causes is unknown.

Dr. Van Bibber showed a specimen of aneurism of the popliteal artery, which had caused the death of a mulatto woman, aged 36 years. The chief interest in the case, apart from the preparation, was the difficulty of diagnosis.

The appearance of the tumor, which was as large as a half gallon measure, was sufficiently indicative of the disease, but the history given by the patient was so at variance with the formation of an aneurism, as to involve the case in doubt. The whole leg was swollen, the skin tense and dry, with deficient sensibility. There was a feeling of deep fluctuation in the tumor, the opening of which was filled with protruded coagulæ and some areolar tissue. Through this opening profuse hemorrhage had taken place, to arrest which, Dr. Van Bibber was summoned. The history was as follows:

In August, a swelling upon the dorsum of the foot was observed; which, by frictions and cataplasms, was relieved. It then extended up the leg, and centred itself in the knee-joint, and by the direction of a physician, had been assiduously poulticed for six weeks before it ruptured. The physician had regarded it as a case of white swelling. The patient was *in extremis* when seen, and simple elevation of the leg arrested the hemorrhage. She sank, however, during the night, notwithstanding stimulants and restoratives of all kinds.

The limb being amputated above and below the knee, the large clots were easily removed with the fingers, showing the effects of the pressure upon the surrounding parts. Plastic deposits had been thrown out in concentric layers of moderate thickness, and had formed firm adhesions; the removal of these showed the opening in the artery, which we suppose to have been originally a true sacculated aneurism. The mouth of the sac is 25 lines in length. The remains of the sac of the aneurism before its rupture, when applied to each other, are in proportion to the size of its mouth, and the continuation of the inner coat of the artery is apparent on the sides of this cavity.

If it were a false sacculated aneurism, the middle coat would terminate abruptly in a thick ring immediately around the mouth and neck of the sac, but it is not so in this case. Moreover, the atheromatous deposits in the tissues of which it is composed, favor this supposition. After the rupture of the true sacculated aneurism, it became a diffused one. The whole duration of the disease seems to have been four months.

Dr. Van Bibber also presented to the society a specimen of fusiform dilatation of the arch of the aorta, with a small sac in the intra-pericardial portion of that vessel. It occurred in a muscular and seemingly healthy negro, and the rupture occurred during a violent exertion. On making an autopsy, the opening in the sac was found to be not larger than the point of a needle, and was only detected by pouring water into the vessel. Death was not so immediate as is usual in aneurismal ruptures into the pericardium, and this was due to the minuteness of the orifice.

Dr. Roby presented a specimen of cancerous disease of the stomach, in which the ulceration had occurred at the superior part of the great curvature; had penetrated the diaphragm, and formed an adhesion to the lung, so that the lower lobe of the lung formed the upper wall of the stomach at the ulcerated part, which was of a circular form, and about two inches in diameter.

Dr. Roby knew nothing of the history of the case, as he had obtained the specimen from the dissecting room of the college.

Dr. Frick presented some specimens of fatty degeneration, with the following history:

Andrew P***, black, æt. 21, entered the prison three years ago. His health had been and was then good, and he had no predisposition to phthisis. In March, 1853, a year after entrance, he had pneumonia of right lung, which confined him to the hospital for three weeks, and from which he never entirely recovered. He was, however, put to light work, and got on quite well until January, 1855, when he again entered the hospital, where he remained till his death.

During this interval, he was never free from a certain amount of dyspnœa, with occasional attacks of suffocative cough and frothy expectoration. Auscultation revealed more or less coarse rales, and some indistinctness about the first sound of the heart. The urine was natural in quantity and quality. On his entrance, in January last, his urine was examined, and found to contain a small amount of albumen. He had slight oedma about his feet, which has existed for some months; complains greatly of pain and sense of tightness over the heart; cough and dyspnœa; expectoration mucous and frothy. Coarse mucous rales were heard in all parts of the lungs and the heart, over which there was increased dullness; had a slight blowing sound over the apex, after the first sound; pulse 106.

He gradually grew worse, and on March, 25th, his condition was as follows: Pulse almost imperceptible, breathing 36, with occasionally some rattling in the throat; skin of natural temperature; intelligence blunted, but easily roused; complained of pain in his limbs and difficulty of getting air; eyes deep yellow; cough frequent and painful; expectoration consisting of frothy blood; abdomen very much distended with fluid; legs swollen and œdematous; has passed no urine for 24 hours; about ℥iv were drawn off, of a dark muddy color, containing a small quantity of albumen and epithelium scales. For two weeks past the quantity of urine has been small, not averaging more than ℥viij, and a specimen examined three days since, gave the following results: Sp. gr. 1,020, dark, with a large deposit of urate of soda, colored scarlet by purpurin, cleared up on heating, to deposit afterwards a large amount of albumen.

Under the microscope, a few crystals of uric acid were seen, with some urate of soda, and a few epithelium scales. To-day, the tumefaction of the abdomen is diminished. There is a little dullness on percussion over the right lung near the spine, where the respiration is somewhat blowing, but not well marked. Coarse mucous rales everywhere, most evident at the summit of both lungs. Percussion over the heart shows increased dullness; sounds feeble and indistinct; some blowing after first sound, most evident over the apex of the heart. His urine, passed the next day, presented the appearance of defibrinated blood. Sp. gr. 1,020, amount, ℥vj. On heating, a very large quantity of albumen was detected. The microscope shows, 1st, many blood globules; 2d, spherical epithelium cells filled with fatty matter; 3d, epithelium cells without fat; 4th, uric acid in amorphous masses; 5th, some irregular bodies of the shape of the tubuli, containing rounded masses of various shapes and sizes. These may be fibrinous casts, but they are indistinct, and the more doubtful from the fact of their not having been observed before. He died on the evening of the same day, March 26th, from suffocation; his intelligence good, and no evidences of poisoning being present. The day before his death, Dr. F. caused him to breathe into a vessel containing hydrochloric acid, but there was no evidence that the expired air contained any carbonate of ammonia, to which it is admitted such poisoning is due, it being produced by the decomposition of urea.

*Autopsy* 12 *hours after death.* Body not emaciated; legs and abdomen much swollen; the latter containing a large quantity of straw colored serum. No evidences of peritonitis; the intestines and head not examined; heart, one-half greater than natural size, and mitral valve strictured to such a degree as scarcely to allow the passage of a common sized lead pencil. Both sides of the heart were filled with fluid blood. Lungs everywhere adherent to the pleura; mucous membrane of the bronchial tubes thick and reddened, the tubes being filled with frothy blood and mucus; posterior part of both lungs much engorged, and middle lobe of right lung hepatized to the extent of about three square inches. In the upper lobe of the right lung were numerous rounded patches, from the size of a pin's head to that of a large pea, not softened, but making the lung friable at this part, and having somewhat the appearance of tubercle; spleen natural in size and appearance; liver natural in size, but resembled nutmeg, was much gorged with blood, and had a greasy feel; right kidney appeared natural externally; the left lobulated and irregular; internally, both were engorged with blood of a deep yellow color, and in both, the encroachment of the cortical substance was very manifest.

The several organs mentioned were all examined microscopically, and gave evidence that fatty degeneration had taken place in a greater or less degree in all. In the *lungs*, the bodies mentioned as resembling tubercle, were found to be composed of spherical epithelium filled with fat globules. In the *heart*, the muscular fibre, though distinct, had fat globules, mostly arranged in lines along and within the muscular fasciculi. The acini of the *liver* were everywhere filled with fat, to a greater extent than the other organs. A portion of the cortical substance of the *kidney* showed large quantities of fat, both within the tubuli and in the epithelial cells. These latter were filled with minute fat vesicles, and were similar to those observed in the urine passed the day before death.

*Remarks.*—This case presents many points of interest, independent of the post-mortem appearance of the kidneys, and their relation to the symptoms during life, viz: 1st. The age of the patient—being only 24. 2nd. The character of the expectoration, indicating pulmonary apoplexy, of which the autopsy gave no evidence. 3rd. The slight blowing murmur after the first sound of the heart, coincident with such an amount of stricture of the mitral valve.

The question as to the relation between the urinary sediment, as revealed by the microscope in those affections included under the name of Bright's disease, and the condition of the kidney which produces them, is one which has engaged attention for the last nine years.

Dr. George Johnson, looking very properly upon Bright's disease as fatty degeneration, declared that the appearnce of oil globules, partly free, and partly contained in epithelial cells which have escaped from the urinary tubes, is the most important evidence of this disease. He has since modified his opinion, and now thinks that five or six different conditions, all attended with the presence of albumen in the urine, may be clearly diagnosed by the urinary sediment—such as chronic and desquamative nephritis, &c. But this has not been found to hold good in practice, and it is probable that, although there are different conditions of the kidney characterized by albuminous urine, they are not yet cleaily distinguished; and in Dr. Johnson's different diseases, we see but different stages of the same disease. For instance, the tubular casts so relied upon as evidencing fatty degeneration, and as being constantly present in this disease, may or may not be found in the urine. They evidently consist of fibrin coagulated in the tubes and retaining their form, having within them albumen, blood globules, &c., and are only evidences of an effusion of liquor sanguinis having taken place into the tubuli uriniferi. Now, this would happen from any cause which tended to produce chronic and slow congestion of the kidneys; and of such causes, fatty degeneration, interfering with the circulation of the organs, is the most important and most common.

If the circulation accommodates itself to this by degrees, or the congestion goes on slowly, only serum containing albumen will be effused; so that these casts may or may not be coincident with Bright's disease.

Dr. Jones, on the other hand, is inclined to think that the fatty deposit is always secondary to a previous congestion or inflammation; but as neither of these conditions is considered by Paget and other observers, at all necessary to fatty degeneration in the heart, nerves or blood vessels, we do not see why it should not be so in the kidneys. There are many other points of interest in this relation suggested by the present case, but we must defer them to another time.

Dr. Van Bibber related a diagnosis of an injury of the eye, made by a member of the society, which he thought highly creditable to his reputation for proficiency in this branch of medicine.

On the 3rd of February, a negro girl, aet. 16, applied at Dr. Van Bibber's office at night, for advice concerning the left eye, which was evidently much inflamed. Upon a subsequent examition he failed to detect two very faint marks on the cornea, and a minute opening in the iris near its outer edge, towards the inner canthus, which were observed by Dr. J. Wilkins, who was requested to examine the eye on the 24th, and which he insisted must have been caused by the entrance of some foreign body. This afterwards appeared to have been the case, as the girl then mentioned an accident which had occurred about three weeks before. Some children were exploding percussion caps near her on a child's toy gun, when she suddenly felt a sharp pain, as if something had struck her eye, but this subsided during the night, and gave her no trouble for the next few days. A week afterwards the eye began to inflame, and it was not until two weeks after the accident that she applied to Dr. Van Bibber. She had never mentioned this circumstance to him, and the cause of the inflammation was only established after Dr. Wilkins had detected the solution of continuity in the iris. The sight of the eye is not yet restored, and the prognosis is unfavorable.

Dr. Wilson presented a specimen, which had been six days in alcohol, taken from a man aged 50, who had died in the Almshouse on the 3d instant. It consisted of the stomach, part of the transverse colon, and a portion of the omentum majus and mesentery. There was scirrhus of the former, greatest at the pylorus, and the two latter were studded with small hardened bodies, much resembling tubercles. No tubercle was found in any other cavity, and the doctor enquired, if, in the opinion of the members, it was likely that tubercle and cancer could coexist. Dr. Frick had seen the association, and mentioned a case of Dr. Miltenberger's, which he had examined about a year ago, in which the cancer cells had taken from the uterus and the tubercular granules from the lungs were both manifest under the microscope.

Dr. Wilson also showed a heart, taken from a man who had died suddenly at the Alms-house. It was exhibited with special reference to the question of concentric hypertrophy. In this specimen the left ventricle is thickened and its cavity nearly obliterated, without a corresponding enlargement of the organ; and this condition of things continued after five days maceration in water, thereby diminishing the probability of its resulting from rigor mortis.

Dr. Wilson presented the scapulæ, clavicles, sternum, humerus, radius, ulna, and bones of the hand, being parts of the skeleton of a woman who had died at the Alms-house recently, after having been an inmate of the house for eight years, suffering with chronic rheumatism.

The specimen was accompanied with a written history of the case. The patient declared that she had suffered with no other disease than rheumatism, and the peculiarity of the skeleton was its extreme lightness and the anchylosed condition of all the joints. Owing to her great emaciation, all the animal portions of the bone had been absorbed, leaving only the earthy constituents.

Dr. Van Bibber related a case, which he remarked was singular, from the manner in which the accident occurred, and so far as he knew was unprecedented, as no physician to whom he had related it, had ever witnessed, or met with a similar one on record.

A student in his office was directed to weigh out one grain of the bi-chloride hydrarg.—and taking the phial in which this substance was contained in coarse powder, poured a portion of it on a small piece of binder's board, supposing it to be a piece of paper. After weighing out the required quantity, he attempted to return the remainder to the phial, by bending the card. This sprung in the middle, and with considerable force, threw the caustic into his eyes. The agony suffered was beyond description. The patient immediately plunged his head into a basin of water, keeping the eyes open, and a syringe was then used to wash beneath the lids. Ether was given to assuage the pain, but the suffering during the night and for several succeeding days was excessive. The treatment employed was directed to the prevention of inflammation, and consisted in cups to the temples and linen cloths over the eyes, which were kept wet with cold water. In a week the left eye was sufficiently restored for moderate use, and both were soon recovered entirely. The bichloride hydrarg. does not appear to have so destructive a caustic action as the caustic potassa, from its effects manifested in this case.

Dr. Wilkins related the following case of symblepharosis:

A man, in blasting rock, had the misfortune to receive injury to both eyes, from which resulted an opacity of the cornea, much obscuring the sight of the right eye. The left upper eye-lid became firmly adherent to the conjunctiva below the margin of the cornea, turning the ball forcibly upwards. A probe could be passed from the external angle of the eye, behind the false membrane, some distance into the cul-de-sac of the conjunctiva. By a successful operation, Dr. W. divided the false membrane, and discovered behind the conjunctiva an abscess, containing several small fragments of the rock. On lifting the posterior wall of the abscess, the cornea was discerned, but little injured. The motions of the eye were restored, the separated surfaces healing without renewing their attachment, excepiing near the inner canthus, where a daily incision was required to prevent their re-union,

The cure is now nearly complete, the eye moving with freedom, and vision almost perfect. It is worthy of remark that the tarsal and reflected conjunctiva had not become adherent as far up as the cul-de-sac, and this case establishes the fact that such adhesions may be successfully treated without the occurrence of re-union.

Dr. F. Jenkins read a report of two cases of puepcral fever, showing, as far as two cases can show, the communicability of this disease by the physician, and its connection with erysipelas.

He was attending a case ot erysipelas in one room, and a woman in an adjoining one being confined at this time, he attended as her accoucheur. On the fourth day she had the initiatory chill, and on the eleventh died of well marked puerperal fever. During this time he attended another obstetrical case, and the woman was attacked on the seventh day after labor with puerperal fever, from which, however, she fortunately recovered.

Dr. Palmer related a case ot malposition of the heart on the right side. His attention had been called to the case by Dr. Sinclair, and they had requested Dr. Buckler to see it with them. The heart does not beat as far to the right of the median line as it does to the left, in its normal position. The patient believes he once had pleurisy, but there was no evidence that this was upon the left side, or had any agency in producing the malposition in question.

Dr. Miltenberger wished to call attention to Dr. Reed's method of reducing dislocations by manipulatiens alone. He had used it successfully in two instances. The first case was a large muscular man, with dislocation of the femur into the ischiatic notch. Many violent but fruitless attempts at reduction had been made, and had produced great soreness and distress about the joint. Chloroform was administered, and by means of abduction, rotation, and extension, the dislocation was reduced in less than two minutes.

Dr. Roby asked if the use of chloroform in the reduction of dislocations was general in this city? He advocated its use, and asked what it is that resists the reduction? Granting it to be muscular contraction, the fact that this is overcome by the remedy proposed, renders its value apparent.

Dr. Buckler asked what is it that assists the surgeon in the reduction of a dislocation by manipulation? If muscular contraction is an agent relied on to draw the bone into its natural situation, this assistance is lost under the influence of chloroform.

Dr. Roby thought it would be much easier to lift the bone into its proper position, all muscular power being subdued, than to contend against the powerful rigidity of muscles when contracted.

Dr. Frick presented a specimen of crystals of cystine, under a magnifying power of 220 diameters. This substance, composed of carbon, hydrogen and oxygen, with 27 per cent. of sulphur, was obtained from the urine of a patient suffering with dyspepsia.

Dr. Roby enquired under what circumstances cystine is found in the urine, to which Dr. Frick replied that Dr. Golding Bird thought the scrofulous diathesis the most common predisposing cause. But Dr. F. had not found it so, and thought that as yet we know very little of the circumstances which favor its production. Dr. F. presented also a specimen of crystalized urea, obtained from the urine of a healthy man.

Dr. Buckler presented a portion of pseudo-membrane, which had been formed in the nares of a child, with the following history: About two years ago the patient, six years of age, was brought to him from Alexandria as a case of rheumatism, with swelling and tenderness around the joints, general cachectic and flabby appearance, with a murmur during the valvular action of the heart. He was not able to say whether this depended on inefficiency of the valves, or upon a peculiar condition of the blood.

His attention was drawn to the singular plastic exudation formed in the nares, which exfoliated spontaneously. It formed very rapidly, and when thrown off, left the mucous membrane a little reddened, but otherwise unchanged. This continued to be formed for a long time, notwithstanding repeated escharotic, stimulant, astringent and alterative applications. The child was not improved when it left his care, and he has heard that it has since died, but of what disease he was unable to learn. He had never seen a case like this before or since.

Dr. Roby related a case possessing two points of interest: First. The difficulty of diagnosis. Secondly. The number of specific diseases with which the patient may have been simultaneously affected. The patient was a girl, aged seven years, who certainly had pertussis. On Saturday evening she retired to bed, complaining of lassitude, headache and fever, her pulse being 130. Sunday and Monday the same. On Tuesday, two or three pimples appeared, and in the evening there were more. On Wednesday, an eruption-like rubeola appeared, and lasted several days, the pustules in the mean time declaring themselves distinctly varioloid. Drs. Frick, Thomas, and Sinclair had seen similar instances, and considered them as rubeolous eruption, preceding variolous disease.

*Cholera Infantum.*—Dr. Buckler had heretofore considered cholera infantum entirely a functional disturbance; but since the point had been discussed in this society, concerning the minute anatomy and functions of Peyer's glands or follicles, which he had always found on dissection enlarged in this disease, he was not certain that it might not be an organic lesion. He had consulted on this subject various authorities—as West, Todd and Bowman, and Carpenter. Some of these considered the follicles shut sacs, while others had discerned their open mouths. Might they not be opened at one period of life, and closed at another? At any rate, if organic lesion existed, it was to be looked for in these glands.

Dr. Roby said, concerning the situation and anatomy of Peyer's glands, he would repeat that he could not receive Dr. Carpenter's authority without an investigation.

In the last edition of his Physiology, Dr. Carpenter described these glands as situated on the side of the intestine which is attached to the mesentery. Dr. Roby said that his own observation would warrant the assertion that this was an anatomical blunder—that the glands are situated on the *free* border of the intestine, opposite its attachment to the mesentery.

Dr. Johnson made the following remarks:

These glands of the intestinal canal may be arranged in three groups, according to their anatomical constitution.

1st. The simple mucous pockets or follicles of Lieberkuhn, existing from the pylorus to the anus, with little variation except in size, being somewhat deeper or longer in the thicker mucous membrane of the large intestines.

2d. The racemose glands of Brunner, or those having a branching duct, around the terminal twigs of which are arranged certain vesicular cells, opening directly and without pedicles, but with a wide opening, just as the vesicles of the lungs open into the finer bronchi. These glands occupy the duodenum.

3d. The follicular glands, or those of which the essential element is a completely closed chamber or follicle, composed of a delicately fibrous vascular capsule, with grayish contents, containing granules, and pale cells resembling lymph corpuscles. In the jejunum especially, the follicles occur singly. These are the *isolated follicles*. In the large intestine there are solitary follicles, differing from the others only in their greater size. And finally, in the ilium, occasionally in the jejunum, and very rarely in the duodenum, the follicles are associated in groups or patches, from 20 to 30, or even 60 in number. These are the so called patches of Peyer. Each follicle lies under a pit in the mucous membrane, which does not quite reach the follicle, and therefore is not its duct. The function of Lieberkuhn's glands, is undoubtedly the secretion of mucus. Of Brunner's glands but little can be said, owing to the almost impossibility of isolating their secretion. Much conjecture has been hazarded on the subject, among which may be advanced the supposition that they secrete a fluid similar in its nature and uses to the saliva. The most interesting, in a physiological point of view, are the patches of Peyer, but, unfortunately, of them also but little is positively known, either as to the nature or manner of their secretion. They are always situated along the free side of the intestine, opposite its mesentric attachment. The follicles of the patches are perfectly closed capsules, like those upon the splenic arterioles. If they emptied themselves into the intestine by bursting, ruptured capsules would occasionally be found, which has not yet happened either in man or the lower animals. The most probable idea is that of Bruecke of Vienna, who, finding the follicles closely surrounded by lymphatics, which he was unable to trace into their interior, and discovering a near resemblance in the semifluid contents of their cells, advanced the hypothesis that they may be appendages to the lymphatic system, and be concerned in the secretion of lymph. In fact, the elements of the lymphatic glands are also closed chambers.

Dr. Buckler made the following observations on the treatment of this disease:

During the first stage—say the first 24 hours—when the access of the disease is sudden, the vomiting and purging are almost incessant, with alarming prostration. Acetate of lead in 3 grain doses, with laudanum or morphia; restoratives of all kinds to obviate syncope; sinapisms; small quantities of iced water; light clothing and a cool situation for the patient; and endeavor to obviate collapse by cordials and mild stimulants.

The treatment thus recommended involves a principle which he thought important. The patient, as thus described, is in a state of spurious hydro-cephalus; the blood has stagnated in the brain, from the deficiency or prostration of the circulation; and this practice is to be preferred to that sometimes adopted, of leeches to the temples and pounded ice to the scalp. This latter course of treatment Dr. B. thought injurious. Subsequently, he gives small doses of calomel with chalk; applications of tepid water to the stomach and head; lime water with the nourishment; (and the lime water, if continued, will certainly change the discharges from the grassy green to a more healthy color.)

The question of removal then arises, and the sea shore is to be preferred, if practicable; but any change, even to another house in the same city, or from a house with east and west exposure, to one with north and south, is beneficial.

Dr. Williams said that he had heard Trousseau recommend raw meat to be given to children.

Dr. Miltenberger had had much experience with the raw meat diet. He recommended a beef-steak to be placed on a plate, scraped with a strong knife, and seasoned with salt, when it is often very acceptable to the child.

Dr. Frick advised that it be prepared with distilled water, a few drops of muriatic acid, and calomel, according to Liebig's recipe.

Dr. Van Bibber said that during the past summer he had tried the *carbazotic acid*, when the acute symptoms had subsided, and a tonic seemed to be indicated, and with such success that he could recommend the remedy. He gave one drop of the saturated alcoholic solution, every four or six hours, to a child one year old, and generally associated with oleum marui.—*Virginia Medical Journal.*

